# Accelerated Testing Method for Predicting Long-Term Properties of Carbon Fiber-Reinforced Shape Memory Polymer Composites in a Low Earth Orbit Environment

**DOI:** 10.3390/polym13101628

**Published:** 2021-05-17

**Authors:** Joon-Hyeok Jang, Seok-Bin Hong, Jin-Gyun Kim, Nam-Seo Goo, Woong-Ryeol Yu

**Affiliations:** 1 Department of Materials Science and Engineering and Research Institute of Advanced Materials (RIAM), Seoul National University, Seoul 08826, Korea; jjh0601@snu.ac.kr (J.-H.J.); diwnsvkfdl@snu.ac.kr (S.-B.H.); 2 Department of Mechanical Engineering (Integrated Engineering), Kyung Hee University, Seoul 17104, Korea; jingyun.kim@khu.ac.kr; 3 Department of Advanced Technology Fusion, Division of Interdisciplinary Studies, Konkuk University, Seoul 05029, Korea; nsgoo@konkuk.ac.kr

**Keywords:** acceleration test, long-term durability, mechanical properties, shape memory composites, space environment

## Abstract

Carbon fiber-reinforced shape memory polymer composites (CF-SMPCs) have been researched as a potential next-generation material for aerospace application, due to their lightweight and self-deployable properties. To this end, the mechanical properties of CF-SMPCs, including long-term durability, must be characterized in aerospace environments. In this study, the storage modulus of CF-SMPCs was investigated in a simulation of a low Earth orbit (LEO) environment involving three harsh conditions: high vacuum, and atomic oxygen (AO) and ultraviolet (UV) light exposure. CF-SMPCs in a LEO environment degrade over time due to temperature extremes and matrix erosion by AO. The opposite behavior was observed in our experiments, due to crosslinking induced by AO and UV light exposure in the LEO environment. The effects of the three harsh conditions on the properties of CF-SMPCs were characterized individually, using accelerated tests conducted at various temperatures in a space environment chamber, and were then combined using the time–temperature superposition principle. The long-term mechanical behavior of CF-SMPCs in the LEO environment was then predicted by the linear product of the shift factors obtained from the three accelerated tests. The results also indicated only a slight change in the shape memory performance of the CF-SMPCs.

## 1. Introduction

Due to their lightweight and specific stiffness, advanced polymer composites have been studied widely for deployable and structural materials in space, including low Earth orbit (LEO) space [1,2,3,4]. In particular, shape memory polymer composites (SMPCs) are promising materials for aerospace structures, as they exhibit self-deploying functionality in response to external stimuli [5,6,7,8,9]. Therefore, many studies have been conducted to develop space structures such as antenna [10,11], hinge [3,12], morphing wing [13], and boom [14] structures, using fiber-reinforced SMPCs. On the other hand, the LEO environment can be fatal to polymer-based composites due to the harsh environment including high vacuum conditions, ultraviolet (UV) radiation, atomic oxygen (AO), and micrometeoroid exposure. In the LEO environment, high vacuum state varies with the altitude. The LEO environment is generally in ultra-high vacuum, i.e., lower than 10^−5^ Torr, and its vacuum level increases up to a maximum of 10^−12^ Torr at 6500 km altitude. High vacuum can lead to outgassing of polymer matrix. UV radiation in the LEO environments ranges from 100 nm to 200 nm so that it can break the molecular bonds of the polymer, causing degradation. When a spacecraft orbits with a velocity about 8 km/s, AO flux ranges from 10^14^ to 10^15^ atoms/(cm^2^s), causing the surface erosion and degrading the mechanical properties of the polymer composites. The thermal cycle also has a great influence on the polymer composite. The temperature range in the LEO environment is between −150 °C and 150 °C, and microcracks in the matrix of the composite can be initiated by the difference in the thermal expansion coefficient of the fiber and the matrix during the thermal cycle [15,16,17,18]. For these reasons, the mechanical properties of polymers and polymer composites in a LEO environment have been of great interest [15,16,17,18,19,20,21]. Recently, researches have been conducted on surface coating and reinforcement to protect against UV and AO exposure [22,23,24]. However, the long-term properties and durability of SMPCs in a LEO environment, i.e., for a duration of up to 10 years, have yet to be fully clarified [25]. Because long-term exposure tests in a LEO environment are not routine and can be practically inefficient, most studies have been conducted in the range of up to 100 h (typically several hours to tens of hours) [16,17,18,19,20,21,24].

Accelerated test methods are commonly used to predict long-term properties and durability. In short-duration experiments, accelerated tests are conducted under conditions that are harsher than those of the target environment to project long-term performance [26,27,28,29,30,31,32,33,34,35]. Changes in properties caused by electromagnetic waves, such as UV light, are predicted by examining the material’s response to shorter wavelengths [36] and increasing temperature [37]. In AO tests, the AO flux is often increased artificially to predict durability [38].

The properties of polymer composites are highly dependent on time and temperature; thus, their long-term behavior can be predicted using the time–temperature superposition principle (TTSP). The TTSP provides the means to measure long-term behavior at a specific temperature, by carrying out experiments at a higher temperature and within a shorter time; with regard to polymer composites, molecular motions accelerate as the temperature of the polymer increases [27,28,31,33,34,35]. In particular, measurement of long-term durability of polymer composites are expensive and time-consuming, temperature-frequency dependent dynamic mechanical model was used to minimize these issue [39,40,41]. Using this principle, many studies have been conducted to predict not only the viscoelastic properties of composite materials, but also the mechanical properties. Representatively, Miyano et al. [34,42] conducted research to predict various long-term properties of polymers such as flexural strength, fatigue properties, and creep properties using TTSP.

The responsiveness of polymers to external stimuli increases with temperature. For example, the reactivity of materials to UV light exposure is more pronounced at higher temperatures, resulting in rapid degradation [37]. In addition, given the same source flow rate of argon and oxygen, the AO flux received by the polymer surface increases with the exposure temperature, resulting in an acceleration effect [38]. These reports suggest that AO and UV effects can be accelerated by raising the exposure temperature. An accelerated test designed by our group confirmed the accelerated effects of carbon fiber-reinforced SMPCs (CF-SMPCs) under high vacuum and UV irradiation at higher exposure temperatures; an accelerated test model based on TTSP was applied to quantify the material response, and the effectiveness of the approach was verified [43]. This work was extended to include AO exposure.

In this study, the long-term properties of CF-SMPCs were investigated in a LEO environment, in which the TTSP was applied to characterize harsh high vacuum, UV light and AO exposure conditions under accelerated testing.

## 2. Materials and Methods

### 2.1. Materials and Sample Preparation

Thermosetting SMP was prepared by applying the formulation previously used by our group. Bisphenol A type epoxy (Epofix^®^; Struer, Denmark) was used, with diamine (Jeffamine D-230; Huntsman Corporation, The Woodlands, TX, USA) as the curing agent. Four layers of woven carbon fabric (TI-3101; TEI Fabrics, Taiwan) were used for reinforcement. CF-SMPCs were fabricated with a vacuum-assisted resin transfer molding (VARTM) method which manufacturing process of fiber-reinforced composite using a vacuum to assist resin flow into fiber-reinforcement. Polymer composite specimens were cured at 110 °C for 3 h and removed from the mold used in the VARTM process. Then, specimens were further cured at 80 °C for 2 h for shape stability and thermodynamically stability of switching segment, which play important role in shape memory mechanism. We confirmed that the epoxy polymer composite had cured completely under that curing condition before proceeding with the experiments through DSC analysis in our previous work [43].

### 2.2. Environmental Chamber and Acceleration Test Design

**Figure 1:**
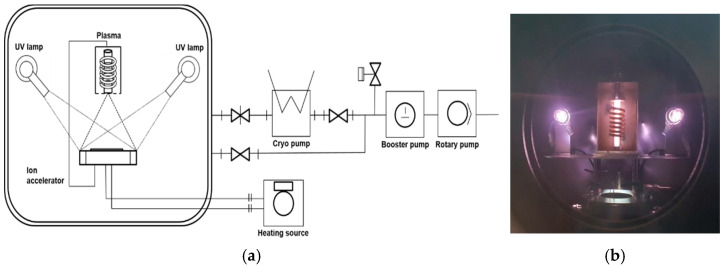

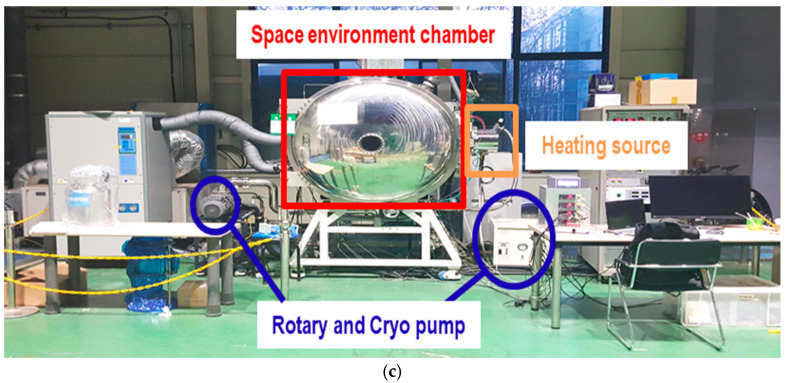
Space environmental chamber: (**a**) schematic diagram, (**b**) built-in chamber, and (**c**) overall appearance.

Figure 1 shows the LEO environmental chamber in our laboratory. The high vacuum system included a rotary pump (W2V10, Woosung, Korea) and a cryogenic pump (Spacetorr, Suzuki Shokan, Japan). The system had a high vacuum atmosphere equivalent to about 10^−6^ Torr. Deuterium lamps (L2D2 lamp, Hamamatsu, Japan) provided UV light over the wavelength range of 190–250 nm. Two lamps were placed on either side of the sample, such that all samples received similar UV exposure. AO experiments used an inductively coupled plasma source, with an ion accelerator to provide a similar AO flux to all samples. AO was generated using Ar and O2, each with a flow rate of 0.8 cc/min, resulting in a plasma power of 0.612 mW. The AO flux in this environment was verified based on the ASTM-E2089 standard [38], in which the AO flux is predicted based on the mass change of the reference material (Kapton^®^ HN; DuPont, Wilmington, DE, USA). The corresponding relationship is as follows:

(1) $$f=\frac{\Delta M}{A\rho Et}$$

where f is the effective AO flux, ΔM is the mass change of the reference materials, A is the exposed area, ρ is the density, E is the erosion yield, and t is the exposure duration. The AO flux was calculated based on the mass loss measured over a 21-h exposure period.

Accelerated tests in AO and LEO environment were designed as follows. In general, the temperature range of the LEO space was −150 °C to 150 °C [16]. In this range, 70 °C was set as the reference temperature, as it resides within the transition region of the epoxy polymer composites. To confirm the acceleration effect of AO on the CF-SMPCs, an environmental exposure test was conducted at three temperatures: 70 °C, 110 °C, and 150 °C. The test proceeded for 21 h. To create high vacuum conditions in the environmental chamber, 3 h was required; thus, a 21 h test was used for experimental convenience. In the case of LEO space with the addition of UV irradiation, UV light and AO simultaneously affected the polymer composite material under high vacuum; for these experiments, the temperature was set to 70 °C, 90 °C, 110 °C, 130 °C, and 150 °C. After each exposure test, shift factors were calculated using the TTSP to obtain a master curve for forecasting the long-term physical properties. In addition, to verify the acceleration test methodology, five new data sets having different times and exposure temperatures, but showing the same effect, were selected and compared.

### 2.3. Characterization

The thermomechanical behavior of the polymer composite specimens was observed to confirm the influence of AO in a LEO environment. The storage modulus and glass transition temperature ($T_{g}$) were measured using a DMA Q800 system (TA Instruments, Inc., New Castle, DE, USA). Three-point bending measurements were conducted, using a span length of 20 mm and multi-frequency strain mode. The temperature range was from 30–150 °C, the heating rate was 5 °C/min, the frequency was 1 Hz, and the amplitude was 15 μm. To quantify the acceleration effect, the samples were characterized with respect to the reference temperature of 70 °C based on the TTSP, over the frequency range of 1–25 Hz. An additional thermal analysis was performed using a simultaneous differential temperature analysis/thermogravimetric analysis (DTA/TGA; SDT650, TA Instruments, New Castle, DE, USA) system. In a nitrogen atmosphere, the heating rate was 10 °C/min, and the measurement temperature range was from 0 to 600 °C. Surface analysis was carried out with secondary electron microscopy (JSM-7600F; JEOL, Ltd., Tokyo, Japan). Chemical analysis was performed using Fourier-transform infrared (FTIR) spectroscopy in attenuated total reflectance mode (Nicolet 6700; Nicolet Instrument Company/Thermo Fisher Scientific, Waltham, MA, USA).

Shape memory performance was characterized under three-point bending thermomechanical cyclic testing [44,45]. First, the temperature was raised above the transition region to form a rubbery state, after which the specimen was deformed by 1.5% strain ($\varepsilon_{i}\to \varepsilon_{d}$). After cooling to a temperature below the transition temperature, the deformation of the specimen was fixed by releasing external loads. Here, the shape fixity ratio ($R_{f})$ was calculated by comparing the fixed strain with the initially imposed strain $\left(\varepsilon_{d}\to \varepsilon_{f}\right)$. Then, the temperature was raised above the transition temperature, allowing for strain recovery ($\varepsilon_{f}\to \varepsilon_{r})$. The recovery ratio ($R_{r})$ was calculated from the strain in the final recovered state, as follows:

(2) $$R_{f}\left(\%\right)=\frac{\varepsilon_{f}}{\varepsilon_{d}}\times 100\;,R_{r}\left(\%\right)=\frac{\varepsilon_{d}-\varepsilon_{r}}{\varepsilon_{d}}\times 100$$

## 3. Results

### 3.1. Effects of AO Irradiation and Temperature on Matrix Erosion

First, AO flux was characterized via the mass change of the reference material (Kapton^®^ HN; erosion yield: 2.81 × 10^−24^ cm^3^/atom) based on ASTM-E2089 [38,46]. Note that the erosion yield of the material represents the volume thereof that is eroded by incident oxygen atoms. The mass loss of the reference film increased with the exposure temperature in the AO environment. Based on the erosion yield of the film, the AO flux in the environmental chamber was determined with respect to the exposure temperature (Table 1), showing the same order of AO flux as that of the space environment (from 10^14^ to 10^15^ atoms·cm^−2^/s) [15,16].

**Figure 2:**
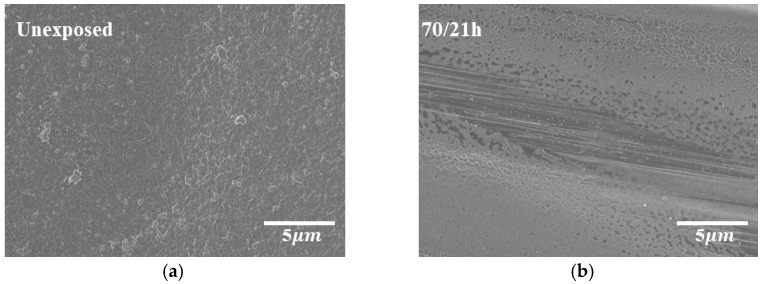

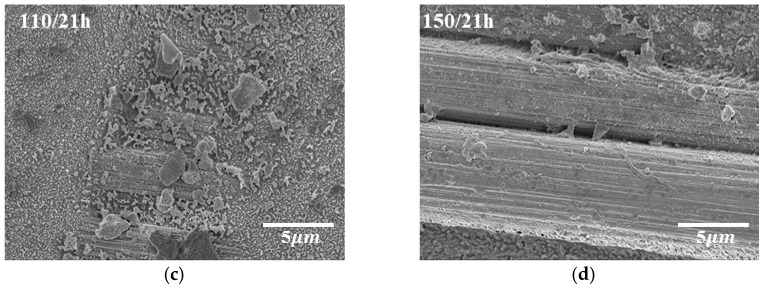
Effects of atomic oxygen (AO) exposure on surface morphology of carbon fiber-reinforced shape memory polymer composites (CF-SMPCs) according to exposure temperature: (**a**) unexposed, (**b**) 70 °C, (**c**) 110 °C and (**d**) 150 °C

**Figure 3:**
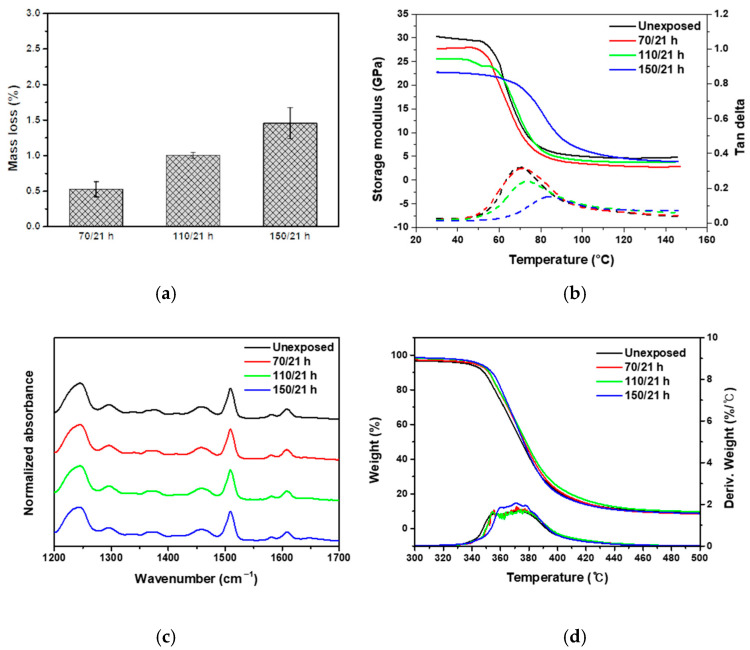
Effects of atomic oxygen (AO) exposure on CF-SMPCs: (**a**) mass loss, (**b**) thermomechanical behavior (straight line—storage modulus, dashed line—tangent delta), (**c**) Fourier transform infrared (FT-IR) spectra and (**d**) thermogravimetric analysis/differential thermal analysis (TGA/DTA) data.

The surface morphology of AO-irradiated CF-SMPCs was observed, and erosion of the SMP matrix was confirmed (Figure 2). It has been reported that carbon fibers also show some erosion when exposed to AO environment [47,48]. In the meantime, other studies showed that, the matrix was mainly affected in carbon fiber-reinforced composites, protecting inner fibers to be less affected [15,16,17,18]. In addition, erosion of matrix accelerated as the exposure temperature increased, i.e., the matrix exhibited severe erosion at 150 °C, compared to samples exposed to 70 °C and 110 °C. This was reflected in the mass loss of the composites. The mass losses of CF-SMPCs were 0.5 %, 1 %, and 1.45 % at exposure temperatures of 70 °C, 110 °C, and 150 °C, respectively (Figure 3a).

The viscoelastic properties of CF-SMPCs before and after AO exposure were measured using dynamic mechanical thermal analysis (DMTA) (Figure 3b, Table 2). The $T_{g}$ of the unexposed sample was 70 °C, and its storage modulus in the glassy region was about 30 GPa. As the exposure temperature increased, $T_{g}$ increased, whereas the storage modulus decreased. In general, the interface between the fiber and matrix is weakened due to the effect of matrix erosion in an AO environment, resulting in degradation of the composite’s mechanical properties [38,49]. In our study, this phenomenon manifested in the storage modulus of the glassy region, in which the apparent degradation accelerated (from 30 to 22.75 GPa) as the exposure temperature increased. On the other hand, the $T_{g}$ of CF-SMPCs increased due to AO exposure. This can be also explained, in part, by the matrix erosion of AO. Due to the erosion of the matrix, the weight fraction of the reinforcing material (i.e., CFs) increased, bringing about a higher $T_{g}$. The reinforcement itself does not contribute directly to the $T_{g}$ change of the matrix; however, the interface between the polymer chain and fiber can significantly affect chain kinetics in the region around the fiber. Therefore, an increase in the weight fraction of reinforcement due to matrix erosion can cause an increase in $T_{g}$ [50,51]. Since the increase in $T_{g}$ was too large to be explained solely by erosion of the matrix, a chemical analysis of the CF-SMPCs was performed.

Chemical changes in the CF-SMPCs according to the exposure temperature were observed before and after AO exposure using FTIR spectroscopy. When using bisphenol A-type epoxy and a diamine-based hardener, three characteristic peaks were observed. The peak at 1250 cm^−1^ is related to the oxirane ring of the epoxy, the peak at 1509 cm^−1^ is associated with the N-H deformation of polyamine, and that of 1509 cm^−1^ corresponds to the C-N stretching vibration peak [52,53]. There was no change in the main characteristic peaks, as shown in Figure 3c, i.e., the functional group did not change after AO exposure. Whether there were chemical changes was inconclusive based on these results alone, as the chemical reactions between functional groups may not be detectable with FTIR spectroscopy [21].

TGA/DTA curves of CF-SMPCs were obtained (Figure 3d). The onset temperature of degradation was characterized according to ASTM E2550 to investigate the change in degree of crosslinking [54]. The onset temperature before AO exposure was 344.98 °C; this increased to 351.67 °C for an exposure temperature of 150 °C (Table 2). These results suggest the possibility of post-curing by AO; in addition, the high energy of AO breaks the molecular chains inside the polymer, and the radicals generated create crosslinks [55]. This post-curing by AO exposure increased the $T_{g}$ of the CF-SMPCs. In summary, AO causes erosion and post-curing of the SMP matrix, which are accelerated by the increased temperature of the AO exposure environment.

### 3.2. Quantitative Analysis of the AO Effect on Long-Term Properties

**Figure 4:**
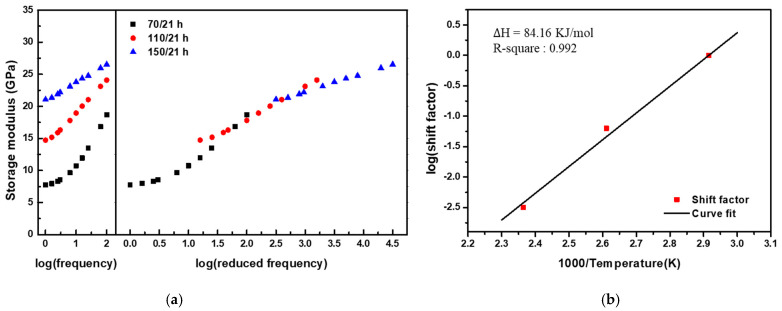
Thermomechanical behavior of AO-exposed CF-SMPCs. (**a**) Storage moduli measured as a function of frequency at a reference temperature of 70 °C using dynamic mechanical thermal analysis (DMA) and the master curve built at the reference temperature of 70 °C, and (**b**) shift factors for the master curve.

Given that the AO effect (matrix erosion and post-curing) accelerates as the temperature of the exposure environment increases, the TTSP, which is commonly used to analyze the long-term properties of polymeric materials, was applied to evaluate the long-term properties of CF-SMPCs exposed to a space environment. In this study, CF-SMPCs were exposed to a high vacuum and AO environment for 21 h at three temperatures (70 °C, 110 °C, and 150 °C). The storage modulus of the CF-SMPCs was measured over the frequency range of 1–25 Hz at a reference temperature of 70 °C (Figure 4a). In Figure 3b, the storage modulus of AO-exposed CF-SMPCs decreased in glassy state, and the decrease was accelerated by exposure temperature mainly due to matrix erosion. In contrast, Figure 4a showed that the storage modulus increased according to exposure temperature. These can be explained by the glass transition temperature of the shape memory polymer (SMP). The reference temperature (70 °C), which is close to the glass transition temperature, was within temperature region where the phase of the SMP transits from the glassy state to the rubbery state. When the exposure temperature of AO increased, not only the matrix erosion but also the post-curing of the matrix due to the energy of the AO irradiation occurred, so the glassy state of the sample exposed to the AO became more dominant at the reference temperature than the unexposed sample. Therefore, despite the occurrence of matrix erosion, the storage modulus increased at the reference.

The long-term properties of CF-SMPCs can be predicted by the TTSP. By simple horizontal shifting the measured data as a function of exposure and time (frequency), a smooth master curve was obtained, from which shift factors could be determined according to the temperature of the AO environment (Figure 4b). This time/environment dependency is described by:

(3) $$G\left(t,T^{AO}\right)=G^{ref}\left(\frac{t}{a_{AO}\left(T\right)},\;T^{AO,ref}\right)$$

where $G^{ref}$ refers to the storage modulus of CF-SMPCs exposed at the reference temperature, $T^{AO,ref}$ is the reference exposure temperature (70 °C), $a_{AO}\left(T\right)$ is the shift factor at the exposure temperature, and G is the predicted storage modulus of CF-SMPCs at the exposure temperature. The shift factor is a quantitative index of how much acceleration has occurred in the actual experimental environment compared to the reference temperature. Experimentally obtained shift factors (Figure 4b) can be expressed using the Arrhenius equation [33,34,42].

(4) $$\log a_{AO}\left(T\right)=\frac{\Delta H_{T,AO}}{2.303R}\left(\frac{1}{T^{AO}}-\frac{1}{T^{AO,ref}}\right)$$

where $\Delta H_{T,AO}$ is the activation energy. The activation energy of the AO process was 84.16 KJ/mol. This shift factor and TTSP were validated by carrying out additional experiments. Here, five conditions, 150 °C/21 h, 139 °C/50 h, 129 °C/100 h, 124 °C/150 h, and 120 °C/200 h, were selected using the Arrhenius equation (Equations (3) and (4)). After the exposure experiment with the selected conditions, the storage modulus and $T_{g}$ of CF-SMPCs were measured through DMTA. Mass loss measurements and TGA/DTA analysis were conducted (Table 3). In this validation test, samples with different fiber orientations from those of acceleration tests (acceleration test: (0/90) orientation and validation test: (+45/−45) orientation) was used. Since the shift factor obtained in the previous acceleration test was mainly due to the post-curing of the polymer matrix, it was necessary to confirm whether this shift factor can be applied to different orientations with the same fiber volume fraction. For this reason, the storage modulus obtained from validation tests was lower than that from acceleration test and the glass transition temperature was also decreased, as reported in a literature [56]. It was confirmed that the prediction model based on acceleration test can be applied to polymer composite regardless of fiber orientation because there was no significant difference the five validation sets. The average $T_{g}$ was 75.50 °C, with a small deviation of about 0.79 °C. In addition, the storage modulus of the glassy region, mass loss, and TGA onset temperature were somewhat similar, confirming the validity of the shift factors and TTSP, as shown in Figure 4.

**Figure 5:**
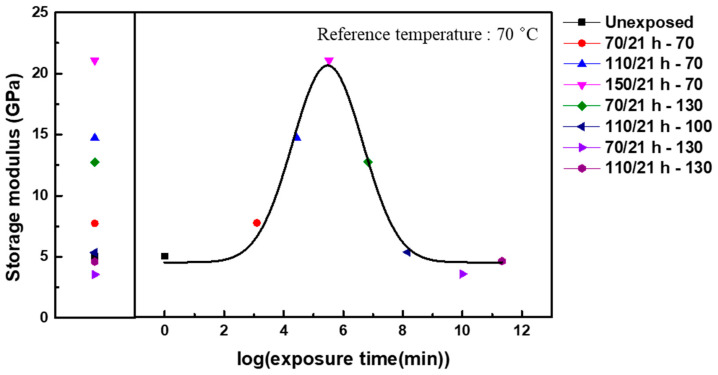
Long-term variation in the storage modulus of CF-SMPCs when maintained at 70 °C under AO irradiation. Legends for each data point represent the AO exposure conditions. The original data were subjected to shift operations.

To predict the long-term properties of CF-SMPCs in a high-vacuum AO environment, the effects of AO should be considered, along with degradation of the polymer due to the time-relaxation process. In addition to obtaining shift factors for accelerated relaxation of polymers over time, our group previously conducted similar tests in a UV environment, proposing a methodology to predict long-term properties based on the linear product of UV and time acceleration factors [43]. In this study, the same concept was utilized to predict the long-term properties of CF-SMPCs in the AO environment, based on the linear product of the shift factors for time-relaxation and AO effects:

(5) $$G\left(t,T,T^{AO}\right)=G^{ref}\left(\frac{t}{a_{T}\left(T\right)a_{AO}\left(T\right)},T^{ref},T^{AO,ref}\right)$$

where $a_{T}\left(T\right)$ is the shift factor of time degradation obtained from previous research. The storage modulus of AO-exposed CF-SMPC samples was measured at various temperatures (Figure 5). The storage modulus of AO-exposed CF-SMPCs increased in the early stage due to the post-curing process. However, after post-curing saturation, the storage modulus decreased due to the erosion and time-relaxation phenomena of the polymer matrix.

### 3.3. Acceleration Effects and Long-Term Properties under LEO Environments

**Figure 6:**
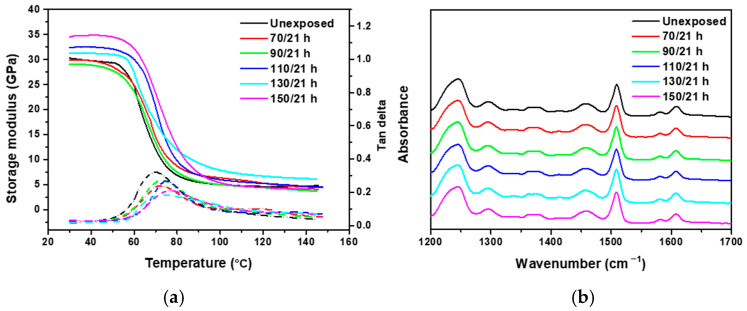

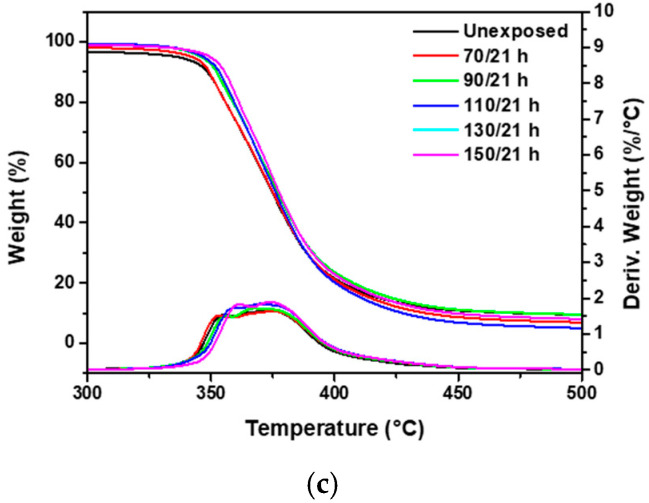
Effects of LEO exposure on CF-SMPCs: (**a**) thermomechanical behavior, (**b**) FT-IR spectra, and (**c**) TGA/DTA curve of LEO-exposed CF-SMPCs.

Accelerated tests were also carried out in the LEO environment under AO and UV conditions. For more rigorous verification than in previous exposure tests conducted in AO environments, the temperature interval was set to 20 °C and exposure tests were conducted at five temperatures (70 °C, 90 °C, 110 °C, 130 °C, and 150 °C). The other experimental conditions were the same as those used in the previous experiments. $T_{g}$ increased, and the increase was more pronounced at higher temperatures (Figure 6a). However, the storage modulus tended to decrease in the AO environment. Notably, the opposite trend was observed in the LEO environment. This was because both UV and AO exposure resulted in the formation of radicals via the breaking of bonds in the polymer matrix, thereby causing post-curing to occur competitively. Thus, the matrix-eroding effect of AO was reduced. Both UV and AO promoted post-curing, which resulted in an increase in the storage modulus. The FTIR spectroscopy results also revealed no change in major peaks (Figure 6b), as in the AO environment, and post-curing was confirmed through TGA analysis (Figure 6c). Through these analyses, the $T_{g}$ of the epoxy-based polymer composite became saturated around 75 °C and did not increase further. Detailed experimental values are summarized in the Supplementary Information section (Table S1).

**Figure 7:**
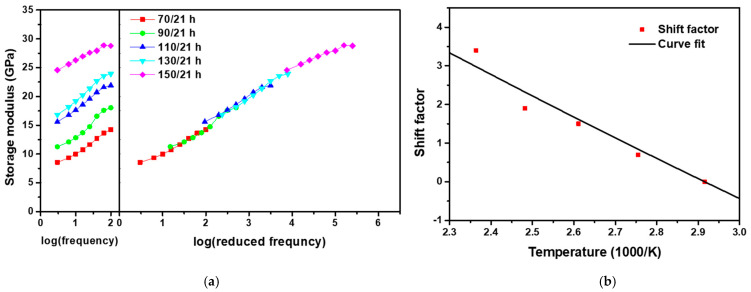
Thermomechanical behavior of LEO-exposed SMPCs. (**a**) Storage moduli measured as a function of frequency at a reference temperature of 70 °C (in DMA) and a master curve built at the reference temperature of 70 °C, and (**b**) shift factors for the master curve.

If there are two competing processes, non-linear Arrhenius behavior can be represented by modifying the coefficients such that two activation energies are considered [57,58]. When such a competitive reaction occurs in an accelerated experiment, a modified Arrhenius equation can be applied that introduces an additional term to represent temperature dependence [59]. In this study, to quantify the acceleration effect in the LEO environment wherein UV and AO are combined, the shift factors of UV and AO obtained through the simple Arrhenius equation were modified with additional coefficients for quantitative analysis. For the experimentally determined shift factor of the LEO environment, as in the previous method, CF-SMPCs were exposed to five temperatures in the LEO space, and the associated storage moduli were measured over the frequency range of 1–25 Hz for the given reference temperature (Figure 7a). A master curve was constructed through the horizontal shifting process of the measured storage modulus. The master curve of the LEO exposed CF-SMPCs showed similar outline to the master curve of the AO-exposed in Figure 4a, but they represent different shift factors. Figure 4 showed a quantified shift factor to show how much the AO effect was accelerated by exposure temperature, while Figure 7 was a quantified shift factor for the acceleration of LEO environment by exposure temperature, which is the linear product of shift factors of UV and AO. As mentioned in Section 3.1, in the transition region, as the exposure temperature increased, the fraction of glass state increased and the storage modulus tended to increase. However, in the LEO environment, unlike in the AO environment, the acceleration effect on UV exposure was also included, so the calculated shift factors were different each other.

The shift factor obtained was analytically fit using the following equation (Figure 7b):

(6) $$\log a_{LEO}=\alpha \log a_{UV}+\beta \log a_{AO}$$

where $a_{UV}$ is the shift factor of the UV environment, which was obtained from our previous work [43], and α and β are the coefficients representing the individual UV and AO processes, respectively. Validation was conducted by comparing five data sets (150 °C/21 h, 139 °C/50 h, 129 °C/100 h, 124 °C/150 h, 120 °C/200 h) that were expected to show the same acceleration effects under different exposure times and temperatures. The measurement results are summarized in Table 4. $T_{g}$, the storage modulus, and the TGA onset temperature showed similar results, supporting the validity of Equation (6).

The storage modulus of CF-SMPCs decreased over time, as described by the master curve and shift factors of time relaxation. However, the LEO environment, which included UV light and AO exposure, gave rise to other behavior, such as post-curing and matrix erosion. We describe these three phenomena (time–temperature relaxation, UV and AO effects in high vacuum) as a linear product of the shift factors which considering them independently. In the current study, we created a prediction model to represent the long-term mechanical properties of CF-SMPCs with long LEO exposure times, by combining the three superposition principles as follows:

(7) $$G\left(t,T,T^{LEO}\right)=G^{ref}\left(\frac{t}{a_{T}\left(T\right)a_{LEO}\left(T\right)},T^{ref},T^{LEO,ref}\right)$$

**Figure 8:**
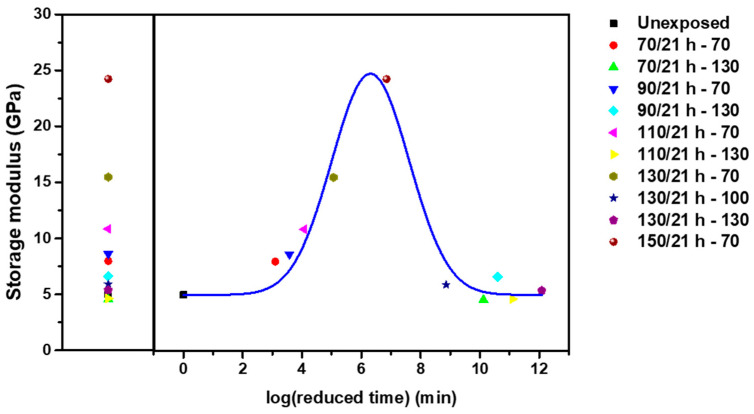
Long-term variation in the storage modulus of CF-SMPCs when maintained at 70 °C under a LEO environment. Legends for each data point represent the LEO-exposure conditions. The original data were subjected to shift operations.

In the same manner as predicted in Section 3.2, a master curve was created by shifting the data with respect to the exposure temperature (Figure 8); a similar master curve is shown for the previous AO environment. The effect of increasing the exposure temperature emphasized the change in properties when the reactions are accompanied by post-curing in the transition region. After exposure, post-curing was dominant in the early part; however, in the second half of the curve, time-relaxation aging and AO-induced degradation were prominent.

We obtained master curves for three environments (UV, AO, and LEO), considering previous research results. In previous our research, as in the AO and LEO environment, UV-induced crosslinking occurred under UV irradiation in high vacuum. The mechanism of this induced crosslinking was different from the thermal curing. In the thermal curing during specimen preparation, the reaction occurred between the epoxide group of main chain and the amine group of crosslink agent. However, UV-induced crosslinking was a newly formed crosslinking during exposure test through the reaction of radicals after chain scissoring by UV irradiation. It was confirmed in our previous work that in UV environment, the storage modulus increased at the initial stage of exposure and then decreased [43].

**Figure 9:**
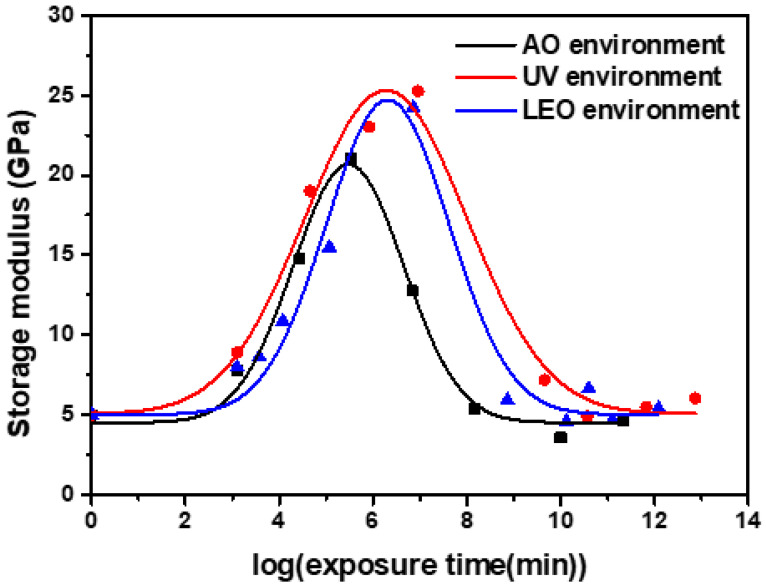
Master curve for each environment (UV, AO and LEO exposure). Each data point represents an experimental value.

In UV, AO, and LEO spaces, the storage modulus increased initially, and subsequently decreased at the reference temperature (Figure 9). However, there was a significant difference in the storage modulus value. In the case of AO, the initial properties increased due to post-curing; however, matrix erosion had only a small effect. The greatest effect was observed with UV exposure; notably, post-curing occurred without any AO effect. In the case of LEO space, the intermediate values between the AO and UV environments reflected the long-term properties under a UV environment.

### 3.4. Shape Memory Properties under AO and LEO Environments

**Figure 10:**
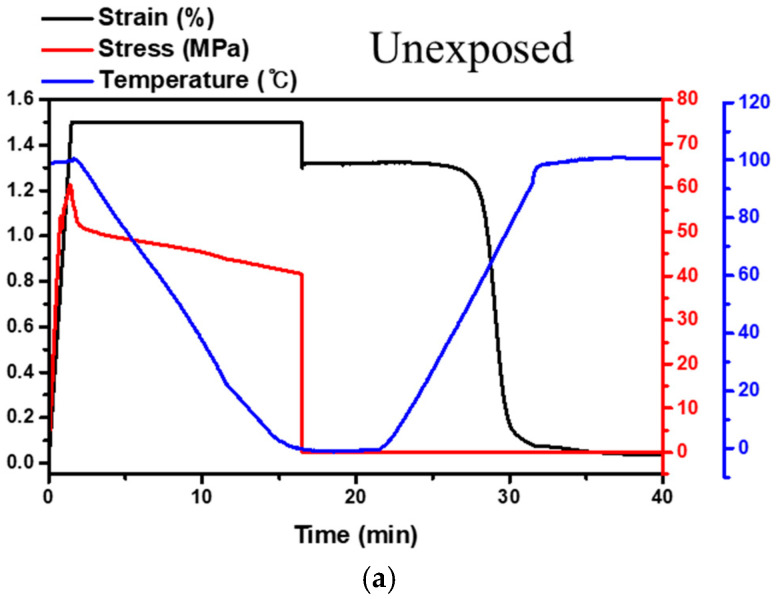

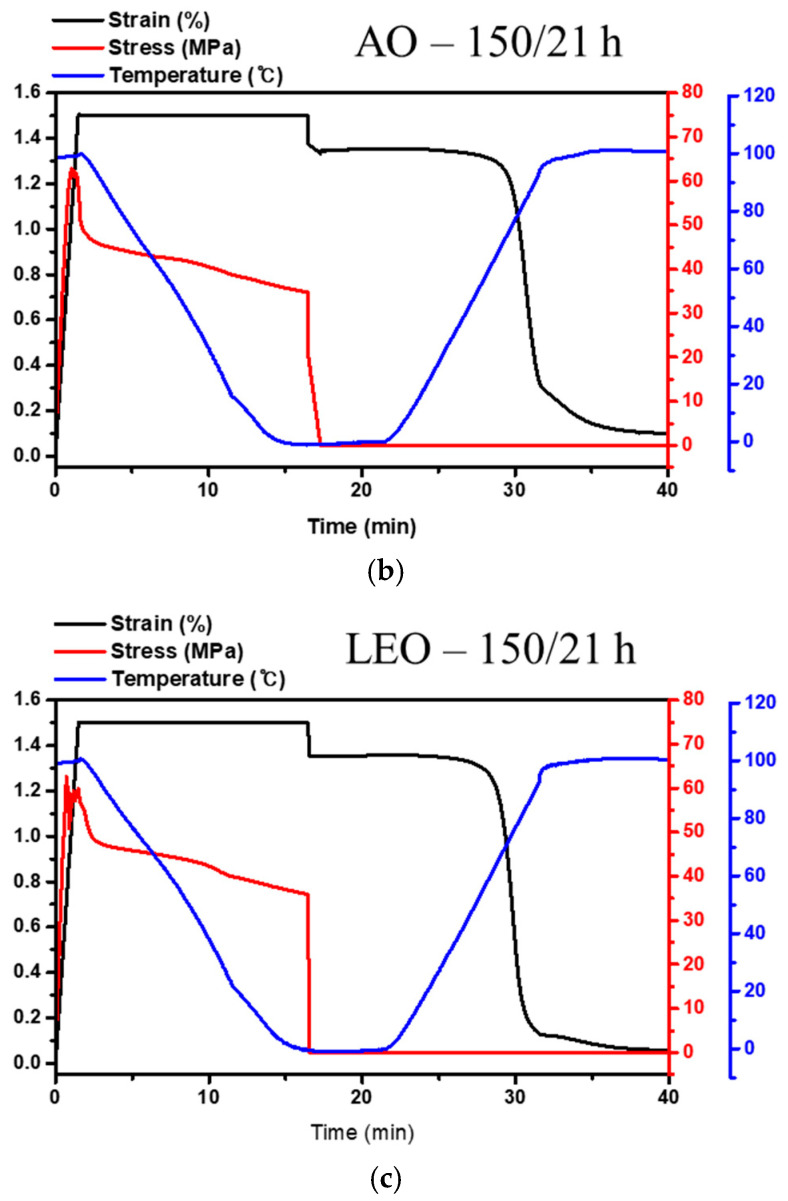
Shape memory properties of CF-SMPCs: (**a**) unexposed, (**b**) 150/21 h under an AO environment, and (**c**) 150/21 h under a LEO environment (for the shape memory properties of AO- and LEO-exposed CF-SMPCs, see Figures S1 and S2 in the Supplementary Information).

The shape memory performance of CF-SMPCs is important for their application to deployable structures in LEO environments. As such, the recovery and fixity ratio of the CF-SMPCs exposed in AO and LEO environments were characterized by a three-point bending thermomechanical cyclic test (Figure 10 and Table 5). There was no significant change in shape memory performance before and after exposure to the AO and LEO environments. The fixity and recovery ratios were about 89% and 97%, respectively, indicating good shape memory behavior. Considering acceleration phenomena in the AO and LEO environments, our results showed that the shape memory performance did not change significantly due to long-term exposure to the AO and LEO environments.

The shape memory performance of epoxy-based thermosetting SMPCs was determined based on the net points of the crosslinking structure and the flexible switching segments. In this epoxy-based material, the crosslink points act as a net point like an anchor, while the main chain of epoxy acts as a flexible switching segment. As such, the number of crosslink (i.e., net points) can determine the shape memory performance [44,60].

Shape memory effect of thermo-responsive shape memory polymer can be explained through entropic elasticity. When the working temperature is higher than the glass transition temperature, the polymer becomes a rubbery state (elastomeric state), and when an external force is applied, it loses its entropy and deforms. At this time, if the temperature is lowered below the glass transition temperature, the flexibility of the switching segment is suppressed by the anchor and the deformed state is maintained. However, when the working temperature becomes higher than the glass transition temperature again, the shape is recovered to its original shape while restoring the lost entropy [7].

In AO and LEO spaces, post-curing of CF-SMPCs progressed and crosslinking increased; however, there was no further change in shape memory performance, as crosslinking had already occurred sufficiently. Note that, as confirmed in Section 3.1, the main functional group of the epoxy-amine-based SMP matrix did not change even after exposure to the AO and LEO environments, which can also explain the unchanged shape memory performance of CF-SMPCs.

## 4. Conclusions

The thermomechanical properties of CF-SMPCs exposed to a LEO environment were characterized using a custom LEO environmental chamber designed to simulate high vacuum and UV and AO exposure conditions. These harsh conditions brought about erosion and post-curing of the polymer matrix, which accelerated as the temperature of the LEO environment increased. The acceleration effect was quantified and modeled using the TTSP. Three shift factors for each acceleration effect (time–temperature relaxation, UV and AO effects in high vacuum) were obtained, and combined based on their linear products, to predict the long-term properties of CF-SMPCs. Finally, the effects of the LEO environment on the shape memory performance of CF-SMPCs were investigated; only slight effects were evident.

## Figures and Tables

**Table 1:** Mass change of Kapton^®^ HN film and atomic oxygen (AO) flux estimation.

Exposure Temperature	70 °C	110 °C	150 °C
Mass change (g)	0.002	0.005	0.008
AO Flux ($\mathrm{atoms}/({\mathrm{cm}}^{2}s)$)	$1.7\times 10^{15}$	$4.1\times 10^{15}$	$6.6\times 10^{15}$
Erosion yield ($E_{y}$)	/	$2.81\times 10^{-24}$	/

**Table 2:** Thermal properties and mass loss of AO-exposed carbon fiber-reinforced shape memory polymer composites (CF-SMPCs).

AO Exposure	Abbreviation	Glass Transition Temperature (°C)	Mass Loss (%)	TGA Onset Temperature (°C)
No treatment	Unexposed	70.00	0	344.98
70 °C/21 h	70/21 h	71.00	0.525 ± (0.107)	349.82
110 °C/21 h	110/21 h	73.50	1.001 ± (0.040)	349.17
150 °C/21 h	150/21 h	84.00	1.454 ± (0.219)	351.67

**Table 3:** Thermomechanical properties and mass loss for five validation data sets in an AO environment.

AO Exposure	Glass Transition Temperature (°C)	Storage Modulus of Glass State (GPa)	Mass Loss (%)	TGA Onset Temperature (°C)
150 °C/21 h	75.00	10.00	1.454	351.67
136 °C/50 h	76.50	10.25	1.119	352.19
124 °C/100 h	75.50	9.78	1.496	352.95
118 °C/150 h	76.00	11.39	1.394	351.65
113 °C/200 h	74.50	8.71	1.165	354.04
Average (Stdev)	75.50 ± (0.79)	10.03 ± (0.96)	1.330 ± (0.17)	352.50 ± (1.01)

**Table 4:** Thermomechanical properties for five validation datasets in a low-Earth orbit (LEO) environment.

LEO Exposure	Glass Transition Temperature (°C)	Storage Modulus of Glass State (GPa)	TGA Onset Temperature (°C)
150 °C/21 h	77.00	34.53	352.39
139 °C/50 h	78.50	33.50	352.80
129 °C/100 h	77.00	35.36	352.60
124 °C/150 h	75.50	34.77	352.58
120 °C/200 h	76.50	36.77	352.83
Average (Stdev)	76.90 ± (1.08)	34.99 ± (1.08)	352.64 ± (0.16)

**Table 5:** Shape memory properties of unexposed and AO and LEO-exposed CF-SMPCs.

AO, LEO—Exposure	Fixity Ratio (%)	Recovery Ratio (%)
Unexposed	88 ± (0.01)	97 ± (0.01)
AO—70 °C/21 h	91 ± (0.56)	97 ± (1.42)
AO—110 °C/21 h	88 ± (0.23)	96 ± (0.40)
AO—150 °C/21 h	89 ± (0.89)	93 ± (0.83)
LEO—70 °C/21 h	90 ± (0.42)	100 ± (0.50)
LEO—110 °C/21 h	91 ± (0.04)	98 ± (0.08)
LEO—150 °C/21 h	90 ± (0.06)	96 ± (0.01)

## Data Availability

The data presented in this study are available on request from the corresponding author.

## Supplementary Materials

The following are available online at https://www.mdpi.com/article/10.3390/polym13101628/s1, Figure S1: Shape memory properties of AO-exposed CF SMPCs: (a) 70/21 h and (b) 110/21 h, Figure S2: Shape memory properties of LEO-exposed CF SMPCs: (a) 70/21 h and (b) 110/21 h, Table S1: Thermal properties of LEO-exposed CF-SMPCs.
