# Supplementary material for: Accelerated Testing Method for Predicting Long-Term Properties of Carbon Fiber-Reinforced Shape Memory Polymer Composites in a Low Earth Orbit Environment
Source: Polymers (Basel). 2021 May 17;13(10):1628. doi: 10.3390/polym13101628 (PMC8156318; doi:10.3390/polym13101628)
Supplement: Supplementary file 1 [file polymers-13-01628-s001.zip › polymers-1201420-supplementary.pdf]

polymers

MDPI

Supplementary information

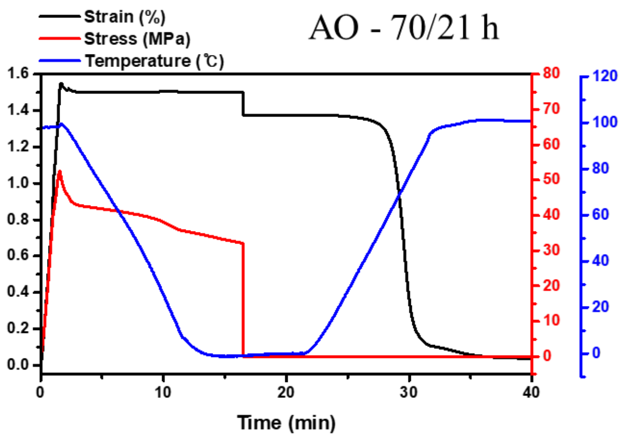

(a)

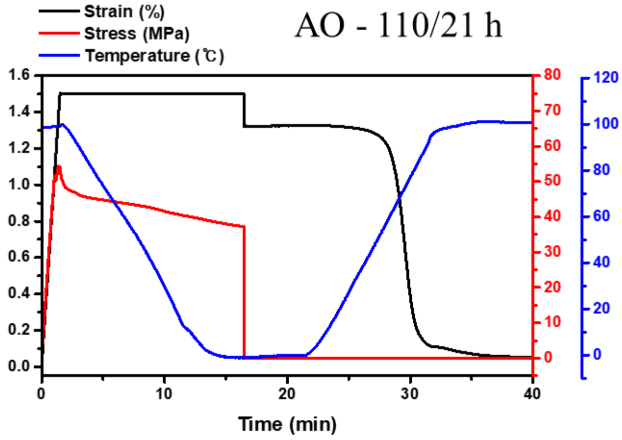

(b)

Figure S1. Shape memory properties of atomic oxygen (AO)-exposed carbon fiber-reinforced shape memory polymer composite(CF-SMPCs): (a) 70/21 h and (b) 110/21 h.

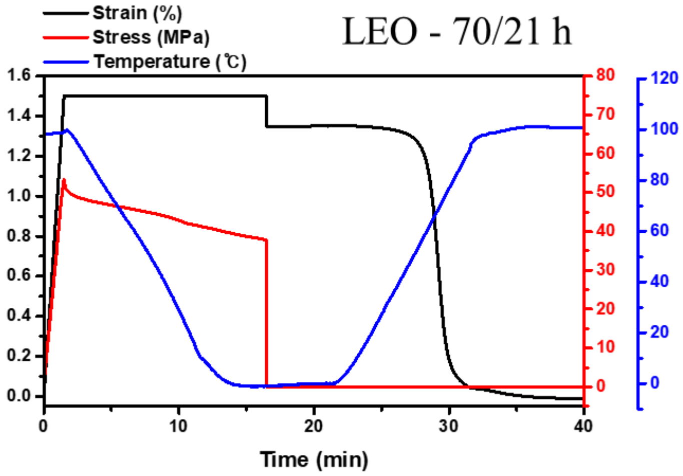

(a)

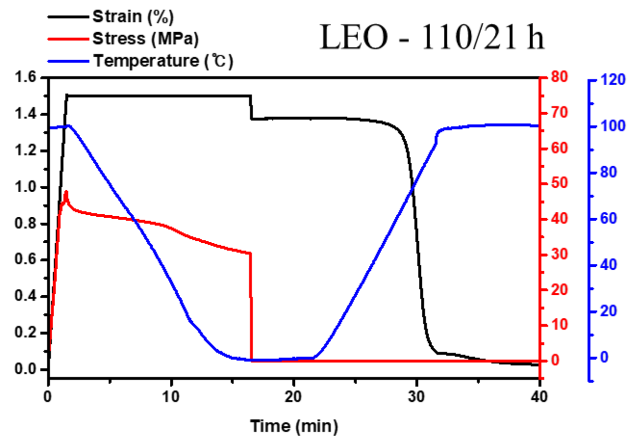

(b)

Figure S2. Shape memory properties of a low Earth orbit (LEO)-exposed CF SMPCs: (a) 70/21 h and (b) 110/21 h.

Table S1. Thermal properties of LEO-exposed CF-SMPCs.

LEO exposure	‘Abbreviation	Storage modulus of glassy state (GPa)	Glass transition temperature (°C)	TGA onset temperature (°C)
No treatment	Unexposed	30.32	70.00	344.98
70°C / 21 h	70/21h	29.92	72.00	344.61
90°C / 21 h	90/21h	29.11	73.00	347.74
110°C / 150 h	110/21h	32.40	72.00	348.92
130°C / 200 h	130/21h	31.25	74.00	347.13
150°C / 200 h	150/21h	34.53	77.00	352.39
